# Geochemical Anomaly Characteristics of Cd in Soils around Abandoned Lime Mines: Evidence from Multiple Technical Methods

**DOI:** 10.3390/molecules26175127

**Published:** 2021-08-24

**Authors:** Lu Wei, Meichen Wang, Guijian Liu, Dun Wu

**Affiliations:** 1 CAS Key Laboratory of Crust Mande Materials and Environment, School of Earth and Space Sciences, University of Science and Technology of China, Hefei 230026, China; weilu101@126.com; 2 Geo-Environment Monitoring Institute of Anhui Province, Hefei 230001, China; 3 State Key Laboratory of Loess and Quaternary of Geology, Institute of Earth Environment, The Chinese Academy of Sciences, Xi’an 710075, China; 4 Exploration Research Institute, Anhui Provincial Bureau of Coal Geology, Hefei 230088, China; mcwang16@163.com; 5 School of Resources and Environmental Engineering, Hefei University of Technology, Hefei 230009, China

**Keywords:** waste lime mine, Cd, mineral composition, spatial distribution characteristics, potential ecological risk assessment

## Abstract

Lime mines are a potential source of pollution, and the surrounding soil environment is generally at threat, especially in abandoned lime mines. This paper focuses on the study area in eastern Anhui, attempting to analyze whether Cd enrichment is related to abandoned mines. On the basis of geological investigation, this study systematically used XRD, XRF, GTS and universal Kriging interpolation to determine the distribution law of Cd in the study area, and evaluated the potential ecological risk of Cd. The results showed that the main mineral types of soil samples of red clastic rock soil parent material (RdcPm) and soil samples of carbonate soil parent material (CPm) were not completely the same. Correlation analysis showed that CaO, MgO and Cd were positively correlated with the CPm. Human activities led to the accumulation of Cd in the study area. High Cd was mainly concentrated in the northwest of the study area, which was correlated with abandoned mines and soil parent materials. The study area was dominated by slight potential risks, although some areas had medium potential risks and high potential risks. All potential high risks were in the CPm field. This study provides a scientific basis for the comprehensive utilization and development planning of soil in the study area.

## 1. Introduction

Ecologically, cadmium (Cd) is one of the most toxic heavy metals, and can have potential adverse effects on soil biological activity, plant metabolism and human health [1]. The naturally occurring Cd in the soil does not generally pose a hazard to humans. However, Cd pollution in soil caused by human activities has serious eco-environmental effects [2,3,4].

According to statistics, in the early 1990s, the area of cultivated land polluted by Cd in China reached 13,000 hm^2^, involving 25 areas in 11 provinces (cities). The area of cultivated land polluted by Cd in China has now reached 280,000 hm^2^, and 7% of the soil Cd concentration exceeds the secondary standard of soil environmental quality, which refers to Cd-polluted soil. Due to rapid economic development, Cd pollution in the ecological environment has become increasingly serious in many areas due to industrial development and mining [5].

Around the world, the environmental safety risks of Cd exposure in soil are being given more and more attention. Due to the chemical properties of Cd and the diversity of pollution sources, it is difficult to conduct scientific investigations on Cd pollution in soil in a region. It is difficult to reflect the concentration and spatial distribution characteristics of Cd in soil objectively and accurately by collecting and investigating small samples. Assessing the distribution of Cd in soil has changed from studying a few samples to the spatial distribution characteristics on a regional scale [6]. Based on multiple geo-accumulation indices and correlation and partial redundancy analyses, Li et al. [7] examined the spatial patterns of agricultural soil contaminations for As, Pb, Cd, Cr, and Ni in the Pearl River Delta, South China, and their relationships with landscape heterogeneity at small, medium and large spatial scales. It was found that distance density variables and land use patterns had stronger influences on trace metal concentrations at a small scale than at a larger scale, whereas the parent materials were important at all the scales. Maas et al. [8] studied the spatial distribution of Cd, Cr, Cu, Pb, and Zn in 101 sub-surface soils, systematically sampled over a large area around Annaba, the fourth most populous city in Algeria. This approach revealed various anthropogenic pollution sources, more efficiently for large-scale patterns than for local abnormalities.

In recent years, with the implementation of geographic information systems (GIS) and Geostatistics (Geostatis-tics, GTS) in environmental research, the spatial distribution of soil heavy metals has been studied using these methods. McGrath et al. [9] used GTS to study the spatial distribution of soil lead and its risk assessment in the Silvemines region of Ireland. Rodríguez-Martin et al. [10] observed the heavy metal concentration of surface soil in Spanish agricultural regions using multivariate GTS. Wang et al. [11] proposed a spatial analysis method based on land use and carried out a human health risk assessment of heavy metals in soil with this method. Dong et al. [12] used GTS methods to calculate the concentrations of As, Hg, Pb, Cu, Cd, Cr and Zn in regenerated soil and at different depths of control soils, and carried out an ecological environmental risk assessment of heavy metals.

In eastern Anhui, China, there are a large number of mined and abandoned lime mines, which may impact the surrounding soil environment. Until now, a large amount of soil pollution has been limited to the study of small samples without background parent material difference, although there are few geochemical studies of soil parent materials considering comprehensively large areas. In this paper, the soil around abandoned lime mines was taken as the research object, and the geochemical characteristics and evolution of soil parent materials with different backgrounds are comprehensively considered. The potential ecological risk of soil was evaluated to provide a scientific theoretical basis for the comprehensive utilization and development planning of soil in the study area.

## 2. Materials and Methods

### 2.1. Sample Collection

The method of collecting surface soil samples in this study is implemented with reference to the relevant methods and technical requirements in the Standards of Geochemistry Evaluation of Land Quality (DZ/T0295-2016), Technical Requirements for Regional Eco-geochemical Evaluation (DD2005-02), and Specifications for Multi-objective Regional Geochemical Investigation (DZ/T 0258-2014).

**Figure 1:**
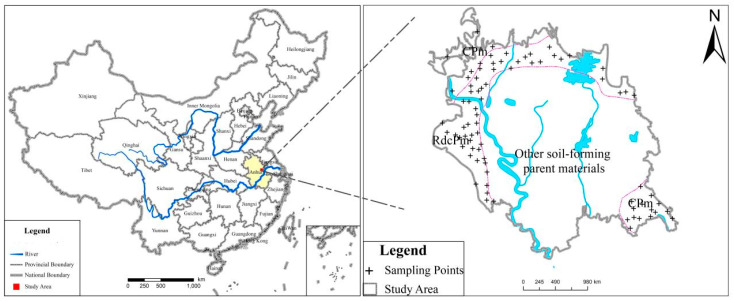
Geographical location of the study area and spatial distribution of sampling points.

Anhui Province is a typical agricultural province in eastern China and is rich in mineral resources. The study area was located in the east of Anhui Province (Figure 1), and belonged to the agricultural area of the Jianghuai undulating plain. The sampling density was 4–16 points per square kilometer, and the basic sampling density was 9 points per square kilometer. The total sampling area was 18.76 km^2^, and 80 surface soil samples were collected in two soil parent material types (RdcPm and CPm).

### 2.2. Sample Preparation

After the collected soil samples were dried and impurities were removed, 500 g of block soil samples was ground and sieved to obtain powder samples with a soil particle size of 200 mesh, and the powder samples were stored for later use.

The abandoned lime mine had a mining history of more than ten years, and it had been abandoned for five years.

### 2.3. Analytical Method

**Figure 2:**
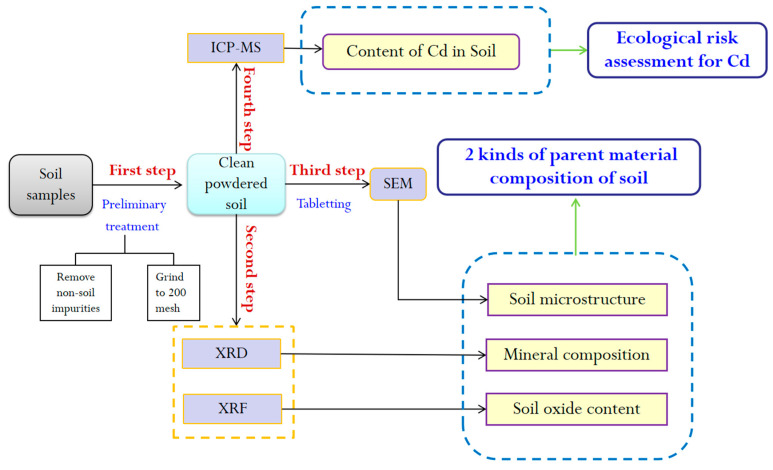
The experimental flow chart of this study. XRD: X-ray diffraction; XRF: X-ray fluorescence; ICP-MS: Inductively coupled plasma mass spectrometry; SEM: Scanning electron microscopy.

The experimental concentrations of this study mainly included the material composition test (XRD and XRF) of soil, soil sample microstructure and cadmium concentration. The purpose of each experiment and the logical relationships between them are shown in Figure 2.

#### 2.3.1. XRD Measurement

An X-ray diffractometer (XRD) was used to test the mineral composition in soil samples. A Philips X’Pert PRO XRD was used to record X-ray intensities scattered from the soil samples. Cu–Kα radiation (30 kV, 160 mA) was used as the X-ray source. Powdered soil samples were packed into a rectangular cavity in an aluminum holder and scanned from 0° to 70° in the 2θ range, with a 0.1° step interval and a 2 s/step counter time.

#### 2.3.2. XRF Measurement

In order to obtain the concentration of the main elements in samples, XRF analysis was performed. The instrument used for XRF analysis of soil samples in this study was XRF-1800 of SHIMADZU Company, Toykoy, Japan. An X-ray tube with Rh target, tube pressure −60 KV (Max), detection element range −4(Be)−(92(U), detection concentration range −10^−6^−100%, minimum analysis area diameter −250 μm was utilized.

#### 2.3.3. SEM Observation

In order to obtain the microstructure of soil samples, a JSM-6490LV scanning electron microscope was used to observe the surface morphology of soil samples. Before observation, Ar ion polishing technology was used to treat the surface of soil samples.

#### 2.3.4. ICP-MS Analysis

In this study, the concentration of Cd in soil samples was determined by inductively coupled plasma mass spectrometry (ICP-MS). A Thermo X Series Ⅱ machine was used, and the working conditions were as follows: transmission power 1250 W, cooling gas flow 13 L/min, auxiliary gas flow 0.75 L/min, carrier gas flow 0.8 L/min, sampling speed 1.5 mL/min, and detector voltage 3850 V. Two standard soil samples (GBW07401 and GBW07402) were prepared for the experiment under the same experimental conditions. The detection limits of the element analysis method are in line with the requirements of the standards, and there was no obvious system error.

#### 2.3.5. Potential Ecological Risk Assessment

The potential ecological risk index ($E_{r}^{i}$) was first proposed by the famous Swedish geochemist Hakanson [13]; it combines multidisciplinary theories such as environmental science, ecology, and biotoxicology. According to the nature of heavy metal elements and the characteristics of geochemical behaviors such as migration and transformation, the potential hazard degree of various heavy metal elements is quantitatively calculated and graded. This method is one of the most widely used methods in the study of heavy metals in international and domestic sediments (soil). Therefore, this study will use the above method to carry out a potential risk assessment of Cd in the soil.

The calculation formulas for the potential ecological risk index ($E_{r}^{i}$) for single heavy metal elements are:

(1) $$E_{r}^{i}=T_{r}^{i}\times C_{f}^{i}=T_{r}^{i}\times C_{n}^{i}/B_{n}^{i}$$

where $T_{r}^{i}$ is the toxicity response coefficient of the heavy metal element i; $C_{f}^{i}$ is the influence coefficient of the heavy metal element i; $C_{n}^{i}$ is the measured value of the metal element i in surface sediment (mg/kg); and $B_{n}^{i}$ is the background reference value (mg/kg) of the metal element i in the sediment. In addition, the toxicity response coefficient of Cd is 30 ($T_{r}^{i}$ = 30) [14].

## 3. Result and Discussion

### 3.1. Determination of Background Values of Two Types of Soil Parent Materials

The background value of soil geochemistry is the most basic parameter used in this study, which represents the changes in the composition, concentration and distribution of materials and elements in different types of soil environments in different regions. The background value of element concentration in soil is a product of the natural geological cycle and biological cycle. The background value is the standard to judge whether the environmental medium (atmosphere, water, soil, etc.) is polluted or not, and to classify the degree of pollution. As for the concept of background value, different subjects give different expressions according to their research objectives and concentrations. In the field of geology, the “Geochemical Background” term was first used in the discipline of exploration geochemistry. The definition of the geochemical background value in classical exploration geochemistry is “the normal abundance of elements in a mineral-free geological body or the normal variation of element concentration in an area” [15,16].

In the field of soil environmental science, the background value of soil geochemistry is the abundance of some original or quasi-original materials or elements in soil in a certain natural historical period in a certain region or statistical unit. Since the Industrial Revolution, the scope of human activities and the degree of impact on nature has been expanding and deepening; the earth’s natural surface media are affected by human activities to varying degrees. Surface soil has become an important sink, continuously accumulating all kinds of pollutants released by human activities. Therefore, the background value of soil geochemistry is an ideal conceptual statistical value, and it is almost impossible to obtain the background concentration of elements by sampling under natural conditions. Based on the above concept, the “soil geochemistry background value” used in this study refers to the concentration of various elements or components in the deep soil (150~180 cm) in the quaternary strata, which is not affected by human activities, and has continuity with the surface soil in soil genesis. The background value is usually characterized by statistical characteristic values (arithmetic or geometric mean value, median and standard deviation) of the element or component concentrations.

**Figure 3:**
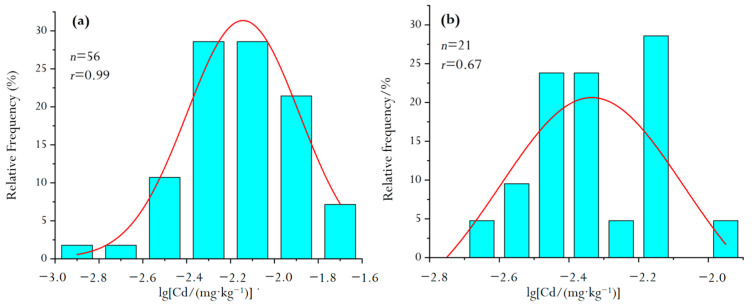
Frequency distribution histogram of Cd concentration in two soil parent materials. ((**a**), RdcPm; (**b**), CPm).

In this study, the total number of soil samples of red clastic rock soil parent material (RdcPm) was 56. The Cd concentrations of the above samples were statistically processed and explained by using normality tests [17]. Results showed that the distribution of Cd was lognormal to the left with a standard deviation of 0.0265 (Figure 3a). The arithmetic mean value (Cd_amv_) of 56 samples was 0.1158 mg/kg and the standard deviation was 0.0264.

The total number of soil samples of carbonate soil parent material (CPm) was 24. The normal distribution of Cd (Figure 3b) could not be obtained by statistical processing of the data and interpretation of the normality test, which indicates that the analyzed sample contains some discrete data. After three cycles the discrete data were eliminated, the remaining samples were reanalyzed, and the results showed that the distribution of Cd was basically normal. The Cd_amv_ of 21 samples was 0.1119 mg/kg and the standard deviation is 0.0171.

### 3.2. Mineral Composition of Two Types of Soil Parent Materials

**Figure 4:**
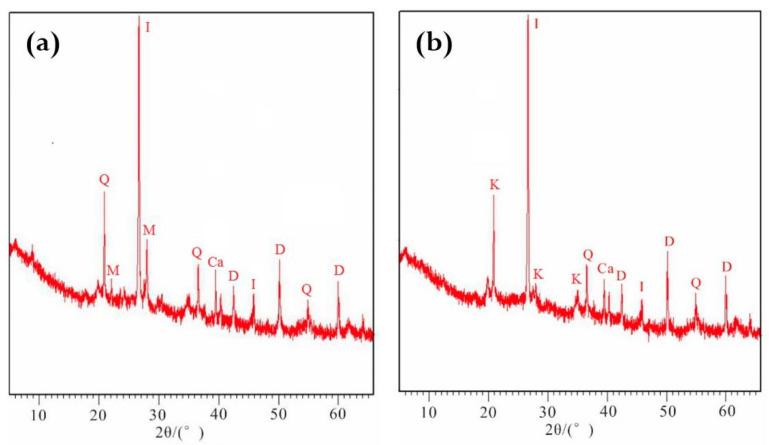
Composition types of minerals in two soil parent materials. (**a**) RdcPm; (**b**), CPm. Q: quartz; M: montmorillonite; I: illite; K: kaolinite; Ca: calcite; D: dolomite.

As can be seen from Figure 4, the main mineral components of RdcPm were quartz, illite and montmorillonite; the main mineral components of CPm were kaolinite, dolomite, quartz, illite and calcite. Using Jade 6.0 software, the proportion of minerals could be calculated by fitting the mineral spectra and calculating the peak area (Table 1). From Table 1, it can be seen that quartz accounts for 36.27% of RdcPm, illite and montmorillonite are the main clay minerals after soil weathering, and carbonate minerals account for 30.8% of CPm; kaolinite is the main clay mineral, followed by illite. The mineral composition results show that the main mineral types of RdcPm and CPm are not identical.

In order to determine whether there was a relationship between the mineral composition and the Cd concentration in the soil, the oxide concentration of the two soil parent materials was determined in this study (Table 2), and the correlations between the concentrations of SiO_2_, MgO, CaO and Cd were determined by correlation analysis (Table 3). The differences in Cd concentrations in soil were mainly caused by the soil mineral compositions. It can be seen from Table 3 that Cd is positively correlated with MgO and CaO (0.01 level) and negatively correlated with SiO_2_ in CPm. However, there was a significant positive correlation (0.05 level) between Cd and MgO, CaO, but not SiO_2_ in RdcPm. The correlation between Cd and MgO, CaO in CPm is more obvious, which is mainly due to the interaction between CO_2_ and water; the main products of carbonate weathering are MgO, and CaO, and precipitated Ca^2+^ precipitates again to form CaCO_3_ [18], and Cd^2+^ can combine with CO_3_^2−^ to form insoluble CdCO_3_.

### 3.3. Microstructure of Two Types of Soil Parent Materials

**Figure 5:**
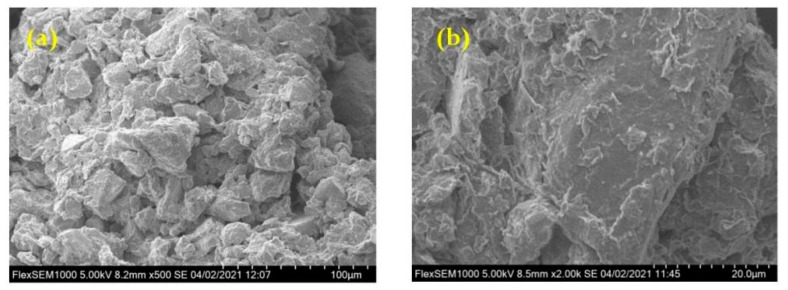
Microstructure characteristics of two soil parent materials: (**a**) RdcPm; (**b**) CPm.

The microstructure of soil influences and controls its engineering geological characteristics [19]. In view of the differences in mineral composition between the above two types of soil samples, it is necessary to further discuss and perform comparative analyses. In this study, scanning electron microscopy (SEM) was used to describe and analyze the microstructure of the samples [20], as shown in Figure 5. Soil samples were mainly composed of small particles and pores, with large pores distributed locally, showing the microstructure of small particles surrounded by large particles. However, the microstructure of RdcPm samples and CPm samples was obviously different. The RdcPm sample soil particles had rough surfaces and sharp edges and corners. However, the particle surfaces of CPm samples were relatively smooth. In addition, in terms of particle compactness, the particle distribution of the CPm sample was obviously looser than that of the RdcPm sample, and the contact mode between particles of CPm sample was mostly surface-to-surface bonding, whereas other contact modes were less close.

### 3.4. Spatial Distribution of Cd in Two Soil Parent Materials

**Figure 6:**
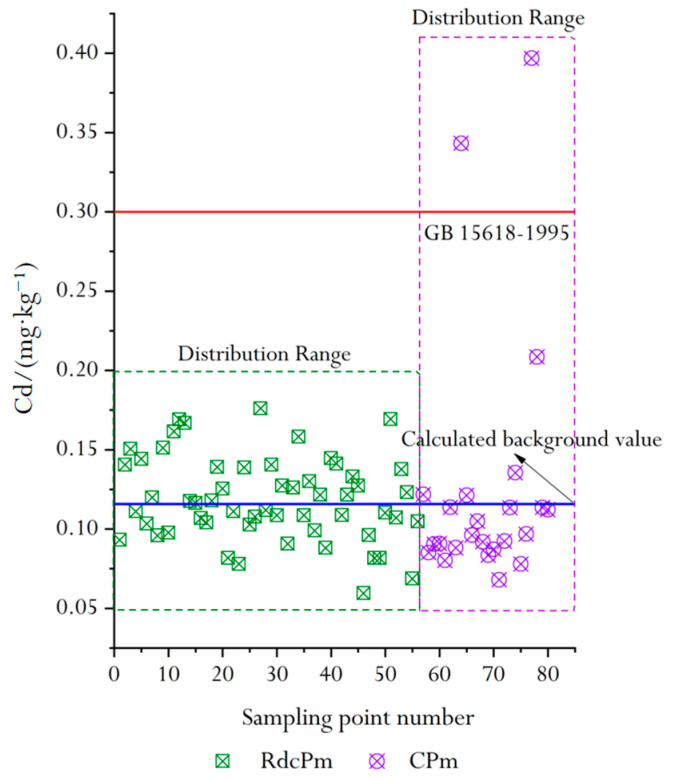
Scatter plot of Cd concentration in two soil parent materials.

The Cd concentration is plotted as a scatter plot in Figure 6. It can be seen from Figure 6 that the Cd concentration of nearly half of the soil samples in the study area was higher than the background value of the study area, which indicates the influence of human activities on natural Cd accumulation [21]. Compared with the Chinese soil secondary standard (GB 15618-1995), the Cd concentration in RdcPm was lower (0.3 mg/kg). However, Cd in CPm was too high locally. This may be due to the concentration of iron and halogen in CPm. This enrichment is not only related to the lithology of carbonate rocks, but also to the strong mineralization of carbonate rock distribution areas [22].

**Figure 7:**
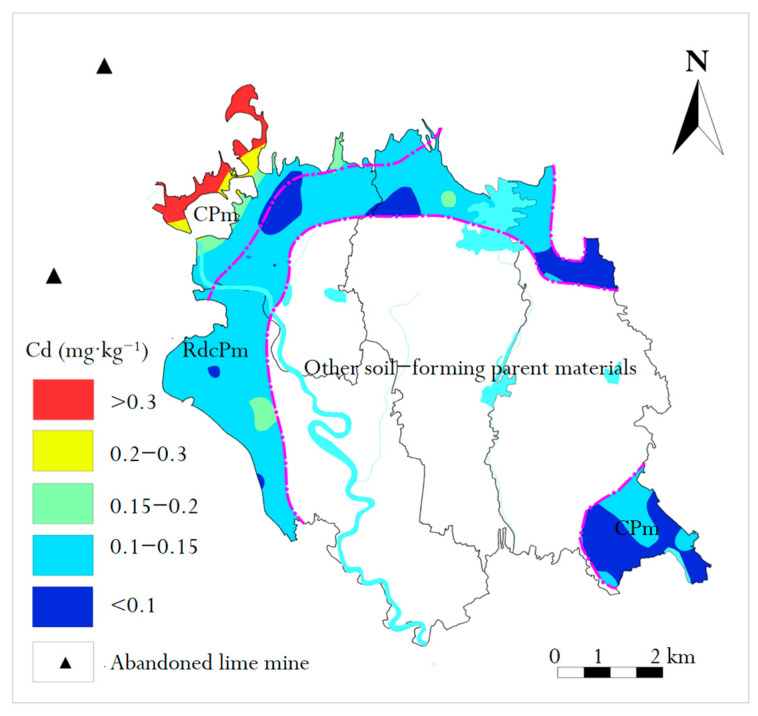
Spatial distribution of Cd in two soil parent materials.

In this study, the background value of Cd in regional soil (0.0992 mg/kg) and the secondary standard of Chinese soil (0.3 mg/kg) were taken as the minimum and maximum values for the spatial distribution (STd) evaluation of Cd in surface soil, respectively. The STd of Cd in the surface soil of the study area was obtained by using the universal Kriging interpolation model [23] in MapGIS software (Figure 7).

The STd of Cd in topsoil of the study area was evaluated according to the Chinese secondary soil standard (GB15618-1995). The results showed that the area of <0.1 mg/kg was 19.82%, 0.1–0.15 mg/kg was 70.20%, 0.15–0.2 mg/kg was 3.89%, 0.2–0.3 mg/kg was 1.55% and >0.3 mg/kg was 4.54%. The concentration of most samples exceeded the background value (0.1158 mg/kg), i.e., the concentration of Cd in most surface soil samples was higher than the background value, which indicated the influence of human activities on the accumulation of Cd in natural soil [24]. Compared with GB15618-1995, the concentration of Cd in the soil in the southeast of the study area did not exceed the standard. The sampling points with greater concentrations than the standard were mainly distributed in the northwest of the research area. It is interesting that the soil parent material type in both regions was CPm. Through site investigation, we found that there were two abandoned open-pit limestone mines in the northwest of the research area. After mining, the mines were abandoned without any environmental protection measures; under certain natural conditions (such as rainfall, wind power, etc.), limestone powder and slag exhibit complex physical chemistry with the surrounding soil, which makes Ca^2+^ and Mg^2+^ chelate with the material in the soil particles, and this geochemistry process may have contributed to the enrichment of Cd. This is consistent with the results of Tarnawczyk et al. [25] and Loredo-Portales et al. [26].

### 3.5. Potential Ecological Risk Assessment of Cd in Two Soil Parent Materials

Based on the analysis of potential ecological risk index ($E_{r}^{i}$), the background value of surface soil Cd in two soil parent materials was used as a reference. The potential ecological risk assessment of Cd on the agricultural quality in surface soil of the study area is presented in Table 4.

According to two types of soil parent materials, the average concentration of Cd in the two regions was between 28.0 and 44.0, indicating that the potential ecological risk to agricultural soil is “slight”. The maximum Cd value of each region had a significant distribution. In the RdcPm region, the potential ecological risk level of Cd reached “middle”. In the CPm region, the value of Cd was more than 200, which reached a “strong” potential ecological risk [27].

**Figure 8:**
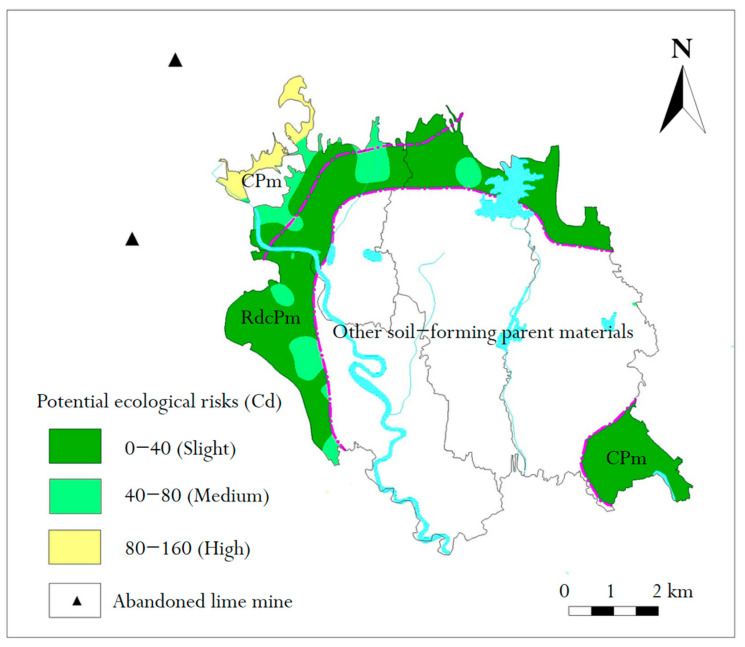
Distribution map of potential ecological risks of two soil parent materials.

In order to further show the proportion of potential ecological risk levels of Cd in two regions, the potential ecological risk index of Cd was interpolated by the universal Kriging method using MapGIS 6.7 software. The spatial potential ecological risk distribution of Cd is shown in Figure 8.

It can be seen from Figure 8 that Cd in RdcPm soil posed a slight risk and middle risk, accounting for 84.65% and 15.35%, respectively. In CPm soil, Cd was mainly a slight risk, accounting for 84.97%; the slight and medium risks of Cd were 7.05% and 7.98%, respectively. The data show that Cd mainly posed a slight potential ecological risk in the study area, and there were a few middle-risk and high-risk areas, whereas high-risk areas only existed in CPm soil (northwest of the study area). On the basis of the above statistical analysis, although the high-risk area was small, there were more medium-risk areas and river systems in the study area. Therefore, it is necessary to study the cause of formation, pollution source and prevention, and control measures of potential ecological risks of Cd in the study area [28]. As soon as possible, abandoned mine ecological rehabilitation should be performed, to control the potential sources of pollution [29]. Strengthening the study of chemical forms of Cd in soil [30], understanding the characteristics of soil geochemistry, and performing the targeted remediation of soil pollution should be the goals of future research.

## 4. Conclusions

The frequency distribution of sample concentrations in the study area were generally normally distributed. The value of Cd_amv_ of 56 RdcPm samples was 0.1158 mg/kg, and the standard deviation was 0.0264. The Cd_amv_ of 21 CPm samples was 0.1119 mg/kg, and the standard deviation was 0.0171.

Soils with different parent materials have similar pore structures. Soil particles of RdcPm samples have rough surfaces and sharp edges and corners. However, the particle surface of CPm samples is relatively smooth.

The Cd concentrations of nearly half of the soil samples in the study area were higher than the background value of the study area, which indicates that human activities cause Cd accumulation. High Cd was mainly concentrated in the northwest of the study area, which was correlated with abandoned mines and soil parent materials.

The study area was dominated by low potential risks, although some areas had medium potential risks and high potential risks. Additionally, all potentially high risks were in the CPm area.

## Figures and Tables

**Table 1:** Mineral compositions of two soil parent materials.

Soil Types	Number of Statistics	Percentage (%)					
		Montmorillonite	Illite	Kaolinite	Quartz	Calcite	Dolomite
RdcPm	*n* = 8	21.90	31.25	NA *	36.27	4.75	5.83
CPm	*n* = 6	NA *	19.24	27.15	22.81	7.68	23.12

NA *: Not analyzed.

**Table 2:** Percentage contents of oxides in two soil parent materials.

Soil Types	Number of Statistics	Percentage (%)				
		SiO_2_	MgO	CaO	MgO + CaO	Other Oxides
RdcPm	*n* = 56	70.23	0.63	0.65	1.28	28.49
CPm	*n* = 24	62.16	1.57	4.81	6.38	31.46

The contents of other oxides were obtained according to 100-(SiO_2_ + CaO + MgO).

**Table 3:** Correlation analysis between Cd element and oxide in soil.

Soil Types	Number of Statistics	Cd	SiO_2_	MgO	CaO
RdcPm	*n* = 56	1	−0.17	0.324	0.328 *
CPm	*n* = 24	1	−0.549 **	0.645 **	0.675 **

** There was a significant correlation at the 0.01 level (bilateral). * There was a significant correlation at the 0.05 level (bilateral).

**Table 4:** Potential ecological risk index ($E_{r}^{i}$) of surface soil Cd in the study area.

Soil Types	$E_{r}^{i}$			
	Min	Max	Ave	Std
RdcPm	16.33	69.90	32.81	9.26
CPm	16.87	206.27	43.43	32.15

## Data Availability

The data presented in this study are available in article.
